# Humanin: a mitochondrial signaling peptide as a biomarker for impaired fasting glucose‐related oxidative stress

**DOI:** 10.14814/phy2.12796

**Published:** 2016-05-12

**Authors:** Anne Voigt, Herbert F. Jelinek

**Affiliations:** ^1^ Department of BiochemistryFreie Universität BerlinBerlinGermany; ^2^ School of Community Health and Centre for Research in Complex SystemsCharles Sturt UniversityBathurstAustralia; ^3^ Division of CardiologyAustralian School of Advanced MedicineMacquarie UniversitySydneyAustralia

**Keywords:** Humanin, impaired fasting glucose, mitochondrial adaptation, mt‐RNR2, oxidative stress

## Abstract

Mitochondrial RNR‐2 (mt‐RNR2, humanin) has been shown to play a role in protecting several types of cells and tissues from the effects of oxidative stress. Humanin (HN) functions through extracellular and intracellular pathways adjusting mitochondrial oxidative phosphorylation and ATP production. Addition of HN improved insulin sensitivity in animal models of diabetes mellitus but no clinical studies have been carried out to measure HN levels in humans associated with hyperglycemia. The plasma levels of HN in participants attending a diabetes complications screening clinic were measured. Clinical history and anthropometric data were obtained from all participants. Plasma levels of HN were measured by a commercial ELISA kit. All data were analyzed applying nonparametric statistics and general linear modeling to correct for age and gender. A significant decrease (*P* = 0.0001) in HN was observed in the impaired fasting glucose (IFG) group (*n* = 23; 204.84 ± 92.87 pg mL ^−1^) compared to control (*n* = 58; 124.3 ± 83.91 pg mL ^−1^) consistent with an adaptive cellular response by HN to a slight increase in BGL.

## Introduction

A link between mitochondrial function and oxidative stress has long been recognized in several disease states including diabetes with the downregulation of electron transport chain proteins and a reduction in oxidative phosphorylation and ATP production (Remor et al. 2011). Mitochondrial‐RNR2 (Humanin) is a member of a class of novel mitochondrial‐derived peptides and released during mitochondrial dysfunction. Humanin (HN) when added to cell culture preparations or animal models of disease may act as a cytoprotective survival factor that has shown promise in protecting against oxidative‐stress‐related diseases (Hill and Van Remmen 2014). It was originally found in a cDNA library survey in surviving occipital lobe neurons of a human Alzheimer disease patient in an open reading frame (ORF) within the mitochondrial 16S ribosomal RNA (Hashimoto et al. 2001; Muzumdar et al. 2009). HN is also found in the kidney, vascular wall, testes, colon, hypothalamus, the heart, and plasma.

**Figure 1:**
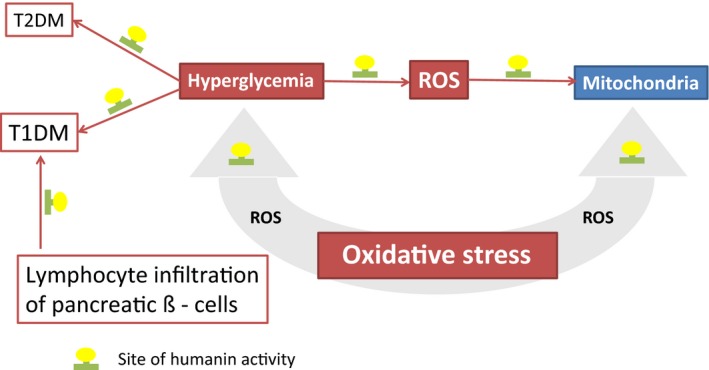
Proposed sites of Humanin action.

Elevated blood glucose levels (hyperglycemia), are a key feature of diabetes, leading to an increase in oxidative stress by increasing free radical activity and mitochondrial dysfunction (Fig. 1) with a further increase in reactive oxygen species (ROS) concentration and damage to endothelial cells in the vascular wall and atherosclerosis (Guo et al. 2003; Rolo and Palmeira 2006). Macrovascular complications are the major cause of death among diabetes patients and are associated with several cellular and biochemical mechanisms within the body's vascular network that have hyperglycemia as a common factor and lead to increased ROS activity, which manifest as oxidative stress and affect mitochondrial function (Maschirow et al. 2015).

Although there is a dearth of human HN studies, several key functions of HN have been proposed based on cell culture and animal models. From these studies, HN has been shown to inhibit apoptosis, rescue mitochondrial function, and improve glucose metabolism by binding proapoptotic, tripartite motif‐containing protein 11 (TRIM‐11), BCL2‐associated X Protein (BAX), and truncated BID (tBid) or through binding to the extracellular ciliary neurotrophic factor receptor/cytokine receptor WSX‐1/glycoprotein 130 receptor (CNTFR/WSX‐1/gp130 receptor) (Kariya et al. 2005; Katayama et al. 2009; Wu et al. 2010; Pagano et al. 2014). Links between HN, diabetes, and diabetes progression have so far been indirect and based on the effects of HN or one of its more potent analogs in cultured cells or animal models. Central infusion of HN or peripherally adding the humanin analog, HN‐GF6A, improved insulin sensitivity and lowered blood glucose levels (BGL) in Zucker diabetic fatty rats, (Muzumdar et al. 2009). HN also improved glucose tolerance and onset of diabetes in a type 1 diabetes mellitus (T1DM) nonobese diabetic (NOD) mouse model (Hoang et al. 2010).

Increased humanin levels as part of clinical studies have been reported for patients with mitochondrial encephalomyopathy, lactic acidosis, and stroke‐like episodes (MELAS) and chronic progressive external ophthalmoplegia (CPEO), which are related to extensive oxidative stress (Kariya et al. 2005). To assess the role of HN in type 2 diabetes mellitus (T2DM) disease progression and its usefulness as a possible biomarker, we analyzed blood samples of participants attending a diabetes complications screening clinic (DiabHealth) (Jelinek et al. 2006).

## Methods

The study was approved by the Charles Sturt University Human Research Ethics Committee. All participants were provided information about the nature of the study and gave written consent. A total of 476 participants were attended the rural diabetes complications screening clinic between 2011 and 2014. To be included in the analysis, participants had to be older than 40 years and clear of cardiac and renal disease as well as hypertension. Gender, age, blood pressure, body mass index (BMI), waist circumference, lipid profile, fasting blood glucose level (BGL), and glycated hemoglobin (HbA1c) were determined for all participants. Blood pressure was measured with a Welsh Allyn clinical sphygmomanometer with an appropriate cuff size to allow for individual differences. Waist circumference was measured with a tape measure to the nearest centimeter and BMI was calculated as the ratio of weight (kg) divided by the height (cm) squared. Lipid profile, BGL, and HbA1c results were provided by the local pathology laboratory after a minimum 12‐hour fast. The participants were divided into a control group (FBG<5.6 mmol/L) and an IFG group (FBG5.6‐ <7 mmol L^−1^) according to the American Diabetes Association Guidelines (American Diabetes Association Position Statement, 2003).

### Biomarkers

Venous blood was collected into ethylenediamine tetraacetic acid (EDTA)‐tubes and the plasma separated by centrifugation for 15 min at 800 *g* and 4°C for HN determination. Additional EDTA tubes were sent to the local pathology laboratory for triglyceride, cholesterol profile, and HbA1c levels. The method suggested by Elisakit.com (Adelaide, Australia) for Humanin Elisa analysis (Lot No. K11064644) was followed. The commercial humanin ELISA kit was previously tested for sensitivity and specificity by the provider. Reproducibility of results on a subgroup of participants indicating a >95% reproducibility with a detection limit of 0.1 ng mL^−1^. Interrater reliability obtained from 20 random samples were as follows: 0.959, 95% CI 0.895–0.984, *P* 0.0001. The Intraclass correlation (ICC) coefficient was estimated as an indication of intrarater reliability and was found to be 0.996 with 95% CI of 0.993–0.998, *P* < 0.0001.

Fasting BGL were determined using the Accu‐Chek^®^ system (Roche Australia). Photometric measurements were carried out with a Thermo Scientific Multiskan FC (Fisher, China). Final concentration measures were obtained with the online program provided by Elisakit.com (http://www.elisaanalysis.com)

### Statistical analysis

Descriptive data are expressed as mean ± standard deviation and analyzed using a Mann–Whitney Test. Chi‐square statistics was applied to identify group membership differences. A general linear analysis was performed to investigate the effect of age and gender on the results. Statistical analysis was performed with SPSS (Version 22, IBM Co). A *P*‐value ≤ 0.05 was considered as significant (Curran‐Everett and Benos 2004).

## Results

Of the 476 participants attending the diabetes complications screening clinic (DiabHealth), blood samples were available for 299 participants. Following exclusion criteria, 81 participants were included in the final analysis, comprising 58 controls and 23 individuals with IFG. The only difference between the control and IFG group detected by chi‐square analysis was in the number of females (Table 1).

**Table 1:** Participant anthropometric and clinical data (mean ± SD)

	Control (*n* = 58)	PreDM (*n* = 23)	*P*‐value
Age (years)	64.1 ± 9.7	65.9 ± 8.9	NS
Females (*n*, %)	40 (69%)	13 (57%)	0.0002
Waist Circumference (cm)	92.1 ± 114	94.2 ± 12.1	NS
BMI (kg/m^2^)	26.2 ± 4.4	26.6 ± 4.5	NS
Supine SBP (mmHg)	122.9 ± 13	133.5 ± 14	NS
Supine DBP (mmHg)	75.21 ± 7.3	78.5 ± 7.2	NS
Medications			
Anti‐HT/antiarrhythmics	0	0	–
Statins	7	4	NS
NSAID	15	9	NS

preDM, prediabetes; BMI, Body Mass Index; SBP, Systolic Blood Pressure; DBP, Diastolic Blood Pressure; AntiHT, antihypertensive medications; NSAID, nonsteroidal anti‐inflammatory medication.

Mean BMI was elevated in both groups and the average supine systolic blood pressure (SBP) was just above normal (>130 mmHg) in the prediabetic group. Both markers were within normal limits.

**Figure 2:**
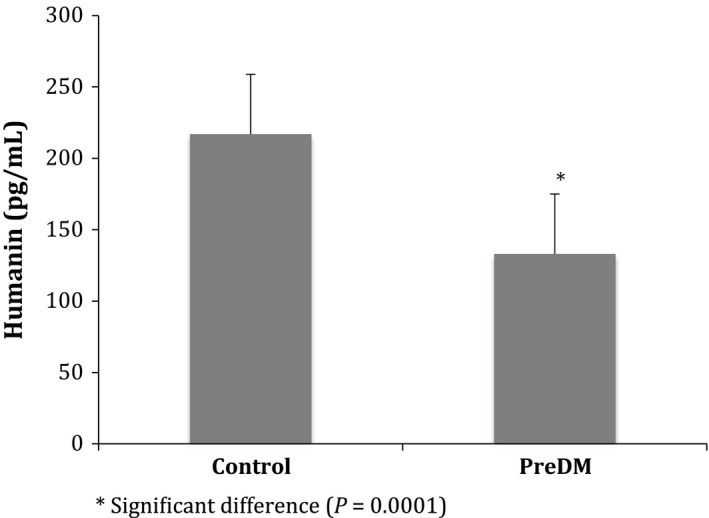
Humanin levels in control and prediabetic participants.

Of the traditional biomarkers only screening glucose levels were significantly different as expected (*P* = 0.0001). Kidney function (estimated glomerular filtration rate, eGFR) and lipids were within normal range. Humanin levels, however, were significantly decreased in the IFG group (Table 2; Fig. 2)

**Table 2:** Biomarkers of control and prediabetes groups

	Control	preDM	*P*‐value
Screening BGL (mmol L^−1^)	4.9 ± 0.5	6.3 ± 0.05	0.0001
eGFR (mL min^−1^ 1.73 m^−2^)	93.2 ± 18.4	99.1 ± 24.7	NS
HbA1c (%)	5.7 ± 0.5	5.7 ± 0.05	NS
TC (mmol L^−1^)	5.28 ± 0.8	5.74 ± 1	NS
Triglyceride (mmol L^−1^)	1.22 ± 0.6	1.3 ± 0.6	NS
HDL (mmol L^−1^)	1.78 ± 0.6	1.55 ± 0.5	NS
LDL (mmol L^−1^)	3.1 ± 0.8	3.3 ± 1	NS
TC/HDL ratio	3.29 ± 1.2	3.73 ± 1.03	NS
Humanin (pg mL^−1^)	204.8 ± 92.9	124.3 ± 83.9	0.0001

preDM, prediabetes; BGL, blood glucose level; eGFR, estimated glomerular filtration rate; HbA1c, glycated hemoglobin; TC, total cholesterol; HDL, high‐density lipoprotein cholesterol; LDL, low‐density lipoprotein; TC/HDL, total cholesterol:high‐density lipoprotein ratio.

**Figure 3:**
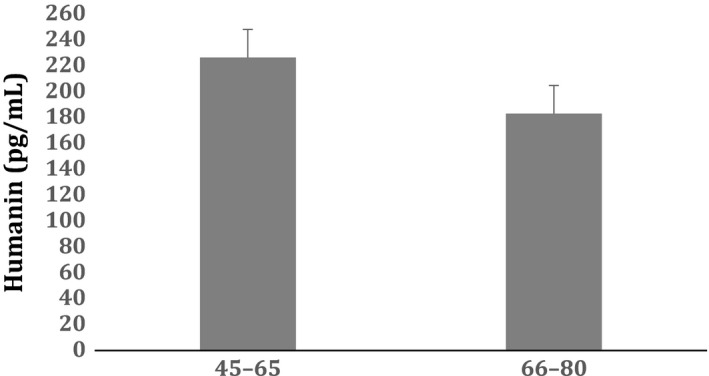
Humanin levels in different age groups.

As HN is associated with oxidative stress we also investigated the effect of obesity, systolic blood pressure,and cholesterol levels by excluding data of participants with BMI >25, SBP >130 mmHg, and a TC:HDL ratio above 3.5. HN still remained significantly lower in the IFG group compared to control. Similar results were obtained when the data were corrected for medication use, which included profilactic use of statins and nonsteroidal anti‐inflammatory (NSAIDs) medication. Age effects on Humanin levels in plasma were compared for a young and older age group of the total cohort (Fig. 3).

Although median HN levels declined across the two age groups (226.5 vs. 183.2 pg mL^−1^) but were not statistically significant.

## Discussion

Our study, using a commercial Humanin ELISA kit, represents the first investigation of HN levels in a clinical population with IFG and showed a decrease in HN levels. Previous work in a diabetic animal model retained better pancreatic islet function (BGL) and insulin sensitivity in NOD mice (Hoang et al. 2010) following humanin supplementation, suggesting that HN is an important protein in inhibiting cell oxidative stress and apoptosis. In advanced disease MELAS and CPEO patients, however, showed noticeably increased plasma HN levels (Kariya et al. 2005; Kin et al. 2006). These findings suggest that humanin has a protective function and is upregulated in disease progression. The decrease in humanin levels with minor increased BGL supports the model of humanin being protective as levels are expected to decrease in response to activation of oxidative‐stress‐associated proteins that are inhibited by humanin.

To ensure that the observed change in HN levels reflected the increased BGL, participants with known diabetes, CVD, and hypertension were excluded. Correction for blood pressure, BMI, waist circumference, and cholesterol levels was not required as there was no difference between the control and IFG groups. We found that HN decreased with age in agreement with previous work but was not significant in our study (Muzumdar et al. 2009). Of note is that in this study HbA1c was not increased in the IFG group above 6.5%, which agrees with previous work of ours in larger cohorts and also with work reported by others. Thus, the acute increased BGL seen in the IFG group already affects the functionality of the mitochondria possibly before there is a change in hemoglobin glycation over time, leading to the observed decrease in humanin as part of its protective function against oxidative stress (Davidson et al. 1999; Mann et al. 2010; Jelinek et al. 2014; Maschirow et al. 2015).

HN has been shown to be effective against oxidative stress in a mouse model of diabetes (Hoang et al. 2010). Injection of HN in nonobese diabetic (NOD) mice revealed a dose‐ dependent protection of neuroendocrine *β*‐cells and delayed, and in some cases prevented the onset of diabetes in this model. HN protects cellular function by increasing glucose tolerance and insulin sensitivity and lowering BGL (Hoang et al. 2010; Gong et al. 2014). An additional proposed mechanism by which HN protects cellular function in IFG is by interacting with hydrogen peroxide and *α*‐actinin‐4, which increase during oxidative stress and IFG (Ha 2006; Francés et al. 2010; do Nascimento et al. 2013). Further proapoptotic TRIM‐11, BAX and tBid, molecules increase in response to oxidative stress in the cytoplasm and bind to HN. Finally HN binds extracellularly with the CNTFR/WSX‐1/gp130 receptor (Kariya et al. 2005; Katayama et al. 2009; Wu et al. 2010; Pagano et al. 2014). A further possible mechanism for mitohormesis was discussed by Long and collaborators, who suggested that mild to moderate levels of ROS lead to positive adaptive mechanisms of the mitochondria and hence could further explain the decrease in HN observed in this study (Long et al. 2014).

All these mechanisms, which may have a beneficial effect on cell function and survival decrease HN levels suggesting a protective function of humanin. With disease progression to T2DM and further oxidative stress, mitochondria may upregulate HN levels as seen in studies of Alzheimer's disease post mortem tissue and in human studies of MELAS and CPEO.

## Conflict of Interest

None declared.
